# Mechanisms of mechanical force in periodontal homeostasis: a review

**DOI:** 10.3389/fimmu.2024.1438726

**Published:** 2024-08-16

**Authors:** Tianqi Wang, Xinran Liu, Jiaxin Li, Yuan Yue, Jinle Li, Min Wang, Na Wei, Liang Hao

**Affiliations:** ^1^ The State Key Laboratory of Oral Diseases & National Clinical Research Center for Oral Diseases, Department of Prosthodontics, West China Hospital of Stomatology, Sichuan University, Chengdu, Sichuan, China; ^2^ The State Key Laboratory of Oral Diseases & National Clinical Research Center for Oral Diseases, Department of General Clinic, West China Hospital of Stomatology, Sichuan University, Chengdu, Sichuan, China; ^3^ The State Key Laboratory of Oral Diseases & National Clinical Research Center for Oral Diseases, Department of Oral Implantology, West China Hospital of Stomatology, Sichuan University, Chengdu, Sichuan, China

**Keywords:** mechanical force, periodontal homeostasis, immune defence, extracellular matrix metabolism, specific proteins, bone metabolism, characteristic periodontal ligament stem cells, non-coding RNAs

## Abstract

Mechanical forces affect periodontal health through multiple mechanisms. Normally, mechanical forces can boost soft and hard tissue metabolism. However, excessive forces may damage the periodontium or result in irreversible inflammation, whereas absence of occlusion forces also leads to tissue atrophy and bone resorption. We systemically searched the PubMed and Web of Science databases and found certain mechanisms of mechanical forces on immune defence, extracellular matrix (ECM) metabolism, specific proteins, bone metabolism, characteristic periodontal ligament stem cells (PDLSCs) and non-coding RNAs (ncRNAs) as these factors contribute to periodontal homeostasis. The immune defence functions change under forces; genes, signalling pathways and proteinases are altered under forces to regulate ECM metabolism; several specific proteins are separately discussed due to their important functions in mechanotransduction and tissue metabolism. Functions of osteocytes, osteoblasts, and osteoclasts are activated to maintain bone homeostasis. Additionally, ncRNAs have the potential to influence gene expression and thereby, modify tissue metabolism. This review summarizes all these mechanisms of mechanical forces on periodontal homeostasis. Identifying the underlying causes, this review provides a new perspective of the mechanisms of force on periodontal health and guides for some new research directions of periodontal homeostasis.

## 1 Introduction

The concept of homeostatic medicine was proposed in 2022 (1). It focuses on the root causes of diseases by exploring the mechanisms and regulation strategies for homeostasis at multiple levels including molecules, cells, organs and systems (2). Under physiological conditions, the periodontal tissue is continuously subjected to environmental stimuli like mechanical forces, salivary flow, and flora stimulation while the internal tissues constantly undergo remodelling to maintain homeostasis (3). The first barrier to the external stimulus is the oral mucosa, which exhibits a strong ability of immune defence (4). Microbiologically, the oral microbiota colonising the mucosal surfaces stimulate a mild immune response and resist invasion of pathogens (4, 5). Next, the extracellular matrix (ECM) metabolism contributes to tissue reconstruction; meanwhile, some mechanosensitive proteins perform functions in response to mechanical stimuli (6, 7). At the cellular level, differentiation between PDL stem cells (PDLSCs) and progenitors could promote tissue maintenance, repair, and regeneration (8, 9). Osteocytes act as an important mechanosensor under mechanical forces (10). Osteoblasts and osteoclasts mediate bone formation and resorption shaping the alveolar bone under mechanical forces (11). At the epigenetic level, some mechanosensing non-coding RNAs, such as microRNAs, could interfere with the expression of related genes (12). In addition, many signal pathways are activated under forces to regulate bone and tissue metabolism (13, 14). These factors help regulate gingiva, periodontal ligament (PDL), and bone homeostasis that form the periodontal homeostasis under mechanical forces.

Mechanical forces including mastication, occlusion, or other forms of mechanical loading exerted on the teeth are transmitted through PDL to the alveolar bone (15). Koivumaa, Mäkilä (16) classified these forces into physiological and non-physiological (pathological and therapeutic) forces. Physiological forces are vital for soft and hard tissue metabolism, which helps maintain periodontal homeostasis. Pathological forces, such as occlusal trauma and bruxism, may damage the periodontium, teeth, or temporomandibular joint. The therapeutic force mainly refers to orthodontic forces that promote tooth movement through bone regeneration and resorption (17). However, absence of physiological forces would result in bone resorption and loss of periodontal tissues (15, 18, 19). Therefore, a balance between proper mechanical forces exerted on the periodontium is quite essential for maintaining tissue homeostasis, whereas improper forces would lead to tissue destruction and breakdown of periodontal homeostasis. There are a variety of mechanosensors on the surface of the cell membrane. After being stimulated by mechanical forces, the mechanosensors undergo configuration changes to activate the cascade signal pathways, and then, biological signals are transmitted to the nucleus to regulate gene transcription (20).

**Figure 1:**
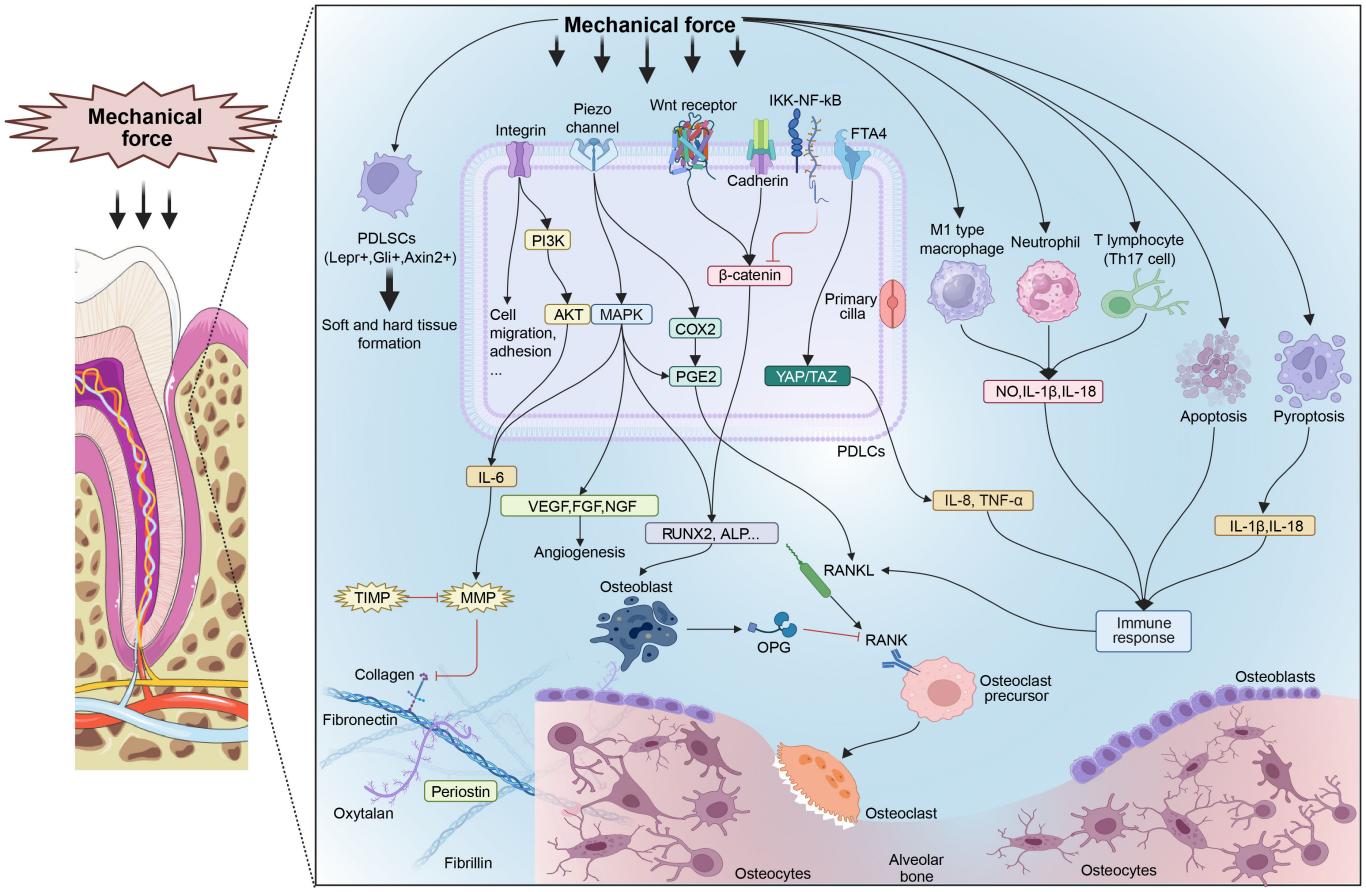
Mechanisms of mechanical forces influencing periodontal homeostasis. ECM components such as collagen, fibronectin, and specific proteins reconstruct under mechanical forces. Several mechanosensitive signal pathways are activated to boost tissue metabolism. Characteristic PDLSCs respond to mechanical forces and promote tissue metabolism. Some pathological forces promote immunocyte differentiation and cell death, leading to an inflammatory response. PDLSCs, periodontal ligament stem cells; PDLCs, periodontal ligament cells; MMP, matrix metalloproteinase; TIMP, tissue inhibitors of metalloproteinases; PI3K, phosphatidylinositol-3-kinase; AKT, protein kinase B; MAPK, mitogen-activated protein kinase; IL, interleukin; VEGF, vascular endothelial growth factor; FGF, fibroblast growth factor; NGF, nerve growth factor; COX2, cyclooxygenase-2; PGE2, prostaglandin E2; Runx2, runt-related transcription factor 2; ALP, alkaline phosphatase; RANKL, receptor activator of NF-κB ligand; RANK, receptor activator of NF-κB; OPG, osteoprotegerin; IKK, inhibitor of IκB kinase; NF-κB, nuclear factor-κ-gene binding; YAP, yes-associated protein; TAZ, transcriptional coactivator with PDZ-binding motif; Th17 cell, helper T17 cell; NO, nitric oxide; TNF, tumor necrosis factor.

This review illustrates the role of forces on periodontal health (**Figure 1**) as well as potential new targets and for homeostatic remodeling which puts forward a new research direction of periodontal homeostasis under forces.

## 2 Aspects of mechanical forces influence periodontal homeostasis

### 2.1 Immune response under mechanical forces

The gingival epithelium acts as a mucosal barrier against external stimuli while commensal microbiota colonising the epithelium resist invasions of pathogens. The microbiota-gingival epithelium barrier contributes to gingiva homeostasis (4, 5). Pathological forces could disturb the balance between microbial communities resulting in breakdown of homeostasis (21). Apart from that, immunocytes in the gingiva also play an important role in maintaining periodontal homeostasis in healthy state (22). Forces have been found to have the potential to affect functions of neutrophils, macrophages, and T cells (23–25). Some improper forces lead to cell death. Specific mechanisms will be illustrated next.

#### 2.1.1 Microbiota - gingival epithelium barrier

Since the gingival epithelium is the first barrier against invasions of pathogens and other stimulations, its integrity and functionality are critical. A previous study has found an ion channel TRPV2 expressed in gingival tissues to sense mechanical stimuli. The ion channel could detect physical and chemical changes to ensure defence mechanisms in gingival tissues (26). Another study also reports the physiological shear stress produced by salivary flow belongs to essential elements of the gingiva. The physiological force is an important guarantee for gingival epithelium to perform immune defence functions (27). Moreover, pathological forces such as occlusal trauma may disrupt balance of microbiological flora and induce infection. Inchingolo et al. found that treating occlusal trauma could relieve the damage caused by the disorganisation of the microenvironment and an increase in pathogenic microorganisms (21). Once the external factors change, such as removal of stress, microenvironment-specific factors, such as temperature, osmotic pressure, and concentrations of metabolites (iron, calcium, and magnesium), will be altered (5). Thus, appropriate forces are essential for the microbiota-gingival epithelium barrier to maintain immune homeostasis.

#### 2.1.2 Immunocytes

As innate immune cells, neutrophils account for the highest proportion of innate immune cells in healthy gingiva, whereas deficiency in neutrophils can increase susceptibility to periodontitis. When exogenous pathogens invade periodontal tissues, neutrophils significantly increase; several steps are required before neutrophils mature and perform functions, including phagocytosis; reactive oxygen species production; and intracellular and extracellular degranulation (22). Granulocyte colony-stimulating factor (G-CSF) may interfere with the CXCR4/SDF-1 axis by reducing chemokine (C-X-C motif) receptor 4 (CXCR4) expression or increasing the stromal cell-derived factor 1 (SDF-1) cleavage and subsequently regulating neutrophil release (28). Traumatic occlusion up-regulates SDF-1 and CXCR4 expression in PDL tissues to maintain bone metabolism by increasing osteoblast differentiation (23). Notably, chemokine (C-X-C motif) ligand 1 (CXCL1), CXCL5, and CXCL8 are responsible for the recruitment of neutrophils. Furthermore, chemokine (C-C motif) motif ligand 2 (CCL2), CCL5, and CXCL10 are chemoattractants of macrophages and lymphocytes. The aforementioned chemokines are regulated by bacterial and mechanical signals. Thus, changes will influence the recruitment of immune cells toward the infection zone (29).

Although fewer macrophages than neutrophils are found in healthy gingiva, they are involved in homeostasis maintenance by phagocytosing pathogens and secreting inflammatory cytokines (30). According to earlier studies, mechanical forces may increase the number of M1-type macrophages and, thus, mediate the inflammatory response and bone remodelling. He et al. found that H_2_S is secreted by PDLSCs under mechanical loads. H_2_S, as a gas signalling molecule, can induce M1-type macrophage polarisation via the STAT1 signalling pathway (31). Jiang et al. found that mechanical force induces PDLSC autophagy. Macrophages are polarised toward the M1 phenotype, led by force-induced autophagy via suppression of the AKT signalling pathway (24).

Healthy gingiva consists of the inflammatory infiltration including T cells acting as antigen-presenting cells (APCs) which regulate local immunity. Balances between helper T (Th) cells including Th1/Th2 cells and Th17/Treg cells are important for the periodontal immune microenvironment (32). Th17 cell differentiates under stimulation by transforming growth factor(TGF)-β and Interleukin (IL)-6 and then releases cytokine IL-17, which is relevant to neutrophil recruitment by activating CXCL8. CXCL8 activation is a key element in periodontal bone resorption, as it facilitates receptor activator of nuclear Kappa-B Ligand(RANKL) production. Similar to Th1/Th2 cells, Th17 and Treg cells perform distinct functions during the inflammatory process. Treg cells generate cytokines, IL-10 and TGF-β, which serve as anti-inflammatory mediators and help maintain immunological homeostasis (33) Recent research has focused on the response of Th17 and Treg cells when exposed to various mechanical forces. Heavy pathological forces up-regulate IL-6, while TGF-β and IL-6 cause Th17 differentiation. In addition, heavy forces promote the expression of HIF-1α, which mediates Th17 differentiation. Consequently, bone resorption and an excessive inflammatory response under heavy force stimulation may be partly attributed to the up-regulation of Th17 cells (25). Mechanisms involved in enhancing the expression of Treg cells and thereby enabling them to perform protective roles in periodontal homeostasis belong to an area of research that remains to be explored.

#### 2.1.3 Cell death

Many reports have suggested that physiological forces influence the metabolism of soft and hard tissues, while pathological forces or improper therapeutic forces result in tissue damage or even cell death (necrosis or programmed cell death). Necrosis refers to the passive death of cells induced by extreme physical or chemical stimuli or severe pathological factors. Once necrosis occurs, membrane permeability increases, resulting in cell swelling; organelle deformation or enlargement; and eventually cell rupture (34).

Programmed cell death is a type of host immune defence that can be disposed due to infected, senescent, or other abnormal cells against infection, radiation, and other harmful situations controlled by genes. Several types of programmed cell death are relevant to periodontal homeostasis under mechanical forces, including apoptosis and pyroptosis. Cysteine-aspartic proteases (caspases) are known to mediate cell death and inflammation during programmed cell death (35, 36).

Apoptosis, a basic physical process, is characterised by cell shrinkage, loss of cell junctions, karyopyknosis, and nuclear fragmentation. The application of mechanical forces can induce PDLSC apoptosis in a time- and force-dependent manner. Regarding fibroblasts, forces from matrix proteins are converted through focal adhesion receptors, which bind to adaptor proteins intracellularly; the adapter proteins link adhesions to the actin cytoskeleton and finally transfer force, thus enabling a cell reaction. Adhesion-associated molecules such as filamin A could prevent cell detachment or detachment-induced cell death under mechanical forces via enhanced formation and maturation of matrix adhesions (37). Piezo1 may transfer force and subsequently activate the p38/ERK1/2 signalling pathway and promote apoptosis in tissue and cells. Furthermore, Piezo1 also acts as a homeostatic sensor that may sense cell crowding, thereby, regulate cell numbers by inducing cell apoptosis or cell division (35). Moreover, the expression of cell caspase-8 and caspase-9, which initiate and execute extrinsic and intrinsic apoptotic pathways, increases under mechanical forces (38).

Pyroptosis, or cell inflammatory death, is an important innate immune process to defend against infection. It is characterised by constant cell swelling and bubbling until the plasma membrane ruptures with the release of cell contents, which induces a strong inflammatory response. Previous studies have found that occlusal trauma and microbial infection can activate the NLRP3 inflammasome and induce pyroptosis in the periodontium, releasing the pro-inflammatory cytokine IL-1β and increasing RANKL expression. In other words, pathological forces cause bone loss and inflammatory responses by inducing pyroptosis. However, physiological forces, such as normal mastication, can induce PDLCs to secrete exosomes, which may interact with macrophages and subsequently reduce IL-1 production. Thus, pyroptosis in macrophages can be reduced by modulating the NF-κB signalling pathway (36). Glyburide, an NLRP3 inhibitor, reduces pyroptosis in periodontal tissues under occlusal trauma and relieves damage. These findings indicate new therapeutic strategies for treating periodontal diseases caused by pathological forces.

### 2.2 ECM metabolism under mechanical forces

The ECM plays an essential role in maintaining tissue integrity and structural stability. Meanwhile, mechanical force is an important stimulus to regulate ECM remodelling, characterised by the synthesis and degradation of matrix proteins, such as collagen type I and III, fibronectin, and laminin, which are generally controlled by matrix metalloproteinases (MMPs) and tissue inhibitors of matrix metalloproteinases (TIMPs) (39). A previous study concluded that mechanical forces significantly impact MMPs and TIMPs in PDL. The precise variation in specific types of MMPs or TIMPs remains elusive, as they are affected by force-applying methods, force-applying time, force magnitude, and other factors. Nevertheless, MMPs generally show a rising trend under mechanical forces, while TIMPs are mainly responsible for controlling MMP activity. For example, TIMPs are up-regulated to reduce MMP activity under tension forces, as the ECM will experience formation and regeneration at this site (6). In addition, a study summarising gene expression changes under mechanical forces showed that genes encoding integrin α5 and αL subunits and their ligands, fibronectin and intracellular cell adhesion molecules, were up-regulated, indicating dynamic mechanotransduction and vigorous cell migration during ECM remodelling (40).

#### 2.2.1 Signalling pathways

Molecularly, mechanical forces could activate some signal pathways or induce cytokine secretion to regulate ECM metabolism. IL-6 is a cytokine up-regulated by mechanical forces and can regulate MMP3 expression through PI3K or mitogen-activated protein kinase (MAPK) signalling pathways (13). Another study found that occlusal forces can activate the sonic hedgehog signalling pathway via Bardet-Biedl syndrome protein-7 and subsequently regulate cell migration and angiogenesis in the PDL (41). Angiogenesis is essential for PDL metabolism, with one study reporting that occlusal forces activate the MAPK signalling cascade to induce the synthesis of vascular endothelial growth factor (VEFG-A), fibroblast growth factor (FGF)-2 and nerve growth factor (NGF), all of which are involved in angiogenesis (22). Another growth factor important for cell proliferation and differentiation, insulin-like growth factor-1, is also induced by mechanical forces via the TGF-β signalling pathway (42).

### 2.3 Specific proteins function under mechanical forces

#### 2.3.1 Collagen, fibronectin and laminin

As the most abundant protein component in ECM, collagen is involved in many biological functions. Some types of collagen form the fibrous structure of gingiva and PDL, which helps to endure mastication forces (43, 44). Besides, type I collagen binding to integrins then transmit extracellular mechanical signals into intracellular signals to mediate cell attachment, proliferation, differentiation, and other functions (7). Fibronectin is the main component of ECM, which plays an important role in cell adhesions. Similar to collagen, fibronectin also contributes to forming PDL fibres. Under mechanical forces, fibronectin combines with integrins to promote cell activity and involves in the cytoskeletal recombination (43, 45). Laminin presents in the epithelial basement membrane that forms a barrier between tissues. Laminin-5 participates in cell-cell interactions mediated by integrins; Laminin-5 is important for cell adhesion, growth, migration, and differentiation (39). The three proteins are all regulated by MMPs and TIMPs that are affected by forces exerted on the ECM (6).

#### 2.3.2 Periostin

Periostin, an important ECM protein, is expressed in the periosteum and the PDL and is crucial for maintaining PDL integrity and stability of the periodontal structure and function, particularly under mechanical forces. Periostin modulates the distribution of ECM proteins, such as fibronectin. In addition, periostin regulates type I collagen fibrogenesis related to the biochemical properties of the PDL (46). Previous research on periostin-null mice found severe periodontal destruction, including sparse and disordered collagen fibres or even loss of collagen fibrous network structure that deteriorated over time after tooth eruption and subjection to occlusal forces (47). A study concluded that the expression of periostin is responsively high in tissues with high mechanical forces and rich collagen. The expression of periostin is up-regulated with increases in the strength of mechanical forces and significantly down-regulated in the absence of mechanical forces (48). Moreover, force-induced periostin could interact with integrin and activate downstream signalling pathways to regulate cell differentiation and migration related to soft and hard tissue formation (49). Under compressive forces during orthodontic processes or some pathological forces, periostin inhibits cell death by regulating Notch 1 expression (50). As discussed above, periostin plays a protective role in maintaining ECM microenvironment stability under mechanical forces. Besides, inflammation may also alter periostin expression, as bacterial or inflammatory stimuli will cause a deficiency in PDL fibroblasts and subsequently reduce periostin production, thus decreasing periostin during periodontitis. Moreover, a lack of periostin leads to increased susceptibility to bacterial infection. Therefore, a decline in periostin destabilises the PDL and aggravates inflammatory infiltration, bone loss, and PDL destruction (51).

#### 2.3.3 Fibrillin

Fibrillin, secreted by periodontal fibroblasts and distributed in the gingiva and periodontal tissues, is a major microfibrillar fundamental element that contributes to connective tissue elasticity and integrity. Microfibrils constitute and participate in forming oxytalan fibres that play a supporting role during mastication. Fibrillin can regulate TGF-β and bone morphogenetic protein activities via interactions with microfibril-associated proteins, such as latent TGF-β-binding protein 1 and fibronectin (52) Fibrillin-1 is also essential for maintaining periodontal homeostasis under mechanical forces and is involved in regulating periostin under mechanical forces via the TGF-β signalling pathway (53). However, another study found no alteration in fibrillin-1 when the PDL was subjected to external forces; therefore, the specific function of fibrillin in periodontal homeostasis remains under exploration.

#### 2.3.4 Integrins

Cell-ECM interaction is an essential process for signal transmission while integrin plays the central role of this process. The integrins are types of transmembrane receptors presented on the plasma membrane link ECM to the cell cytoskeleton, and thereby mediating cell adhesion, migration, mechanotransduction, and other physiological processes. Periodontal cells mainly express integrinα2β1, α3β1, and α5β1, its extracellular domain can bind to ECM components like collagen and fibronectin with high affinity while its intracellular domain is responsible for the recruitment of adaptor protein and scaffold protein and indirect interaction with microfilament cytoskeleton (54, 55). As a force sensor, force applied to the ECM fibre will induce conformational changes of integrins and their cytoplasmic domain bind to actin-binding adaptor proteins that transmit applied forces from integrins to the actin cytoskeleton. The downstream cell activities respond in several ways. One is the recruitment and activation of signalling proteins like FAK, paxillin, SRC and ERK and then activate some downstream signal pathways like PI3K/AKT. The other way connects ECM and integrins directly to the nucleus, enabling force to be transmitted to the nucleus to further regulate gene expression (56).

#### 2.3.5 Focal adhesions

Focal adhesions(FAs), mechanosensitive macropolymers, anchored at junction between cell and the ECM. The focal adhesions composed of several protein layers including the integrin signalling layer; the intermediate force transduction layer containing talin and vinculin; and the microfilament skeleton regulatory layer (55). According to FAs in periodontal tissues, periodontal cells mainly express integrin α2β1, α3β1, and α5β1 that could combine with ECM components including collagen and fibronectin, and then activate downstream signaling crosstalk via proteins such as vinculin or the actin cytoskeleton and downstream effectors (55). This mechanism is essential for periodontal cells to detect and measure the mechanical properties of their microenvironment, such as periodontal tissue stretching and shear stresses exerted by salivary flow, and respond appropriately to these cues (20).

#### 2.3.6 Cadherins

Cadherins are a family of transmembrane glycoproteins that mediate calcium-dependent cell-cell adhesion. Similar to focal adhesion, cadherins are mechanosensitive that have a similar function with focal adhesions. In response to mechanical forces, cell adhesion receptors such as cadherins can induce actin cytoskeletal recombination, which alter cell activity (55); E-cadherin transmits external forces and activates Adenosine 5‘-monophosphate-activated protein kinase (AMPK), showing a protective role against metabolic disturbances (57). Cadherin-11-mediated adherens junctions can alter the mechanical properties of the ECM (58). Another study discovered that cadherin-11 shows decreased expression in a time- and intensity-dependent manner under mechanical force stimulation. Meanwhile, β-catenin expression is altered in conjunction with cadherin-11. Moreover, β-catenin can translocate into the nucleus and induce osteogenesis- and fibrogenesis-related gene expression. In addition, cadherin-11 can mediate ECM collagen synthesis, and cadherin-11 knockdown in PDLCs changes cell shape and suppresses collagen synthesis. Accordingly, a previous study indicated that the cadherin-11/β-catenin pathway in PDLCs is inhibited by mechanical stresses, which may change the shape of the PDLCs and reduce collagen production (59).

#### 2.3.7 Nuclear proteins

Forces are transmitted from ECM via FAs through cytoskeleton to the nucleus from several pathways. First, actin polymerisation directly affects the conformation of nuclear pore complex (NPC), and then the mechanosensitive transcription regulators such as YAP/TAZ (Yes- associated protein/transcriptional coactivator with PDZ- binding motif) and myocardin-related transcription factors (MRTFs) flow inside the nucleus to modulate gene expression (56). Second, stress fibres mechanically connect the ECM and FAs to the nucleus via the linker of nucleoskeleton and cytoskeleton (LINC). LINC is mainly composed of nesprin and SUN-domain protein while nesprin connects outwards to the cytoskeleton such as cytosolic actin, microtubules, and intermediate filaments in the cytoplasm and SUN-domain protein is anchored to the nuclear lamin (60). This yields transcriptional modulation through transcription factors and chromatin changes (61). LINC is mainly composed of nesprin and SUN-domain protein while nesprin connects outwards to the cytoskeleton such as cytosolic actin, microtubules, and intermediate filaments. in the cytoplasm and SUN-domain protein is anchored to the nuclear lamin (60). The interaction between lamins and LINC plays an important role in the regulation of nuclear mechanical properties and mechanotransduction. Lamins could directly regulate chromatin distribution and gene transcription through self-expression, post-translational modification, and structural changes after stress stimulation. Although abnormalities in lamins also impair LINC causing cytoskeleton nucleolysis coupling leading to mechanotransduction defects and downstream chromatin dysfunction (62).

### 2.4 Bone metabolism under mechanical forces

Since the most obvious clinical feature of periodontal destruction is bone resorption, bone metabolism is one of the most important elements in periodontal homeostasis. Osteocytes, derived from osteoblasts, are the most abundant cells in bone. By secreting regulatory factors, osteocytes could regulate the activities of osteoblasts and osteoclasts (63). Most importantly, osteocytes function as the main cell in bone to sense mechanical stimulation to induce mechanotransduction (64). Regarding mechanosensors on the osteocytes, a previous review has reported some mechanosensors including cytoskeletons (actin filaments, microtubules, and intermediate filament); cell dentrites and cell body; primary cilla; integrin-based FAs; gap junctions(Cx43); ion channels; and glycocalyx (10). The downstream signalling pathways that are activated to regulate bone metabolism will be discussed in the next paragraph. Bone formation and resorption are executed by osteoblasts and osteoclasts to keep alveolar bone homeostasis. Physiological forces, such as mastication and normal occlusion, boost circulation and metabolism of periodontal tissues, while loss of occlusion leads to atrophy of periodontal tissues and bone resorption (19). Simple pathological forces such as traumatic occlusion cause vertical resorption of the alveolar ridge and negatively impact tooth mobility but do not damage gingival tissues by forming periodontal pockets or cause attachment loss. In some circumstances, a tooth subjected to pathological forces can shift or tilt toward the compression side to eliminate applied forces. This mechanism is similar to orthodontic tooth movement. Therapeutic forces such as orthodontic forces exerted on the tooth surface can promote tooth movement through alveolar bone reconstruction. Normally, bone resorption happens on the compression side, and bone formation occurs on the tension side (17).

#### 2.4.1 Signalling pathways

The mechanosensitive signalling pathways may control gene transcription and protein expression, leading to bone formation and resorption. For instance, on the compressive side, compressive forces induce an aseptic inflammatory response to generate inflammatory cytokines, such as IL-1β, IL-6, and tumour necrosis factor (TNF)-α, thus stimulating RANKL expression and osteoclastogenesis. On the tension side, osteoblastogenic factors, such as alkaline phosphatase (ALP), runt-related transcription factor 2 (Runx2), bone morphogenetic protein (BMP), osteocalcin, and type I collagen, are found to be up-regulated in PDL fibroblasts (14). The canonical Wnt pathway is essential in osteocytes for mechanosensing and regulating bone mass, while its specific role in osteoblastogenesis or osteoclastogenesis depends on the force type, force application time, and force magnitude. Canonical ligands, such as Wnt1, Wnt3a, and Wnt10b, increase under mechanical loading (65). Moreover, the non-canonical Wnt pathway regulates bone metabolism, and Wnt4 promotes bone formation by inhibiting IKK-NF-kB and activating the WNT-PCP-ROCK pathway under conditions of periodontitis and occlusal trauma (66). Additionally, Wnt pathway inhibitors, such as sclerostin, dickkopf 1, and Wnt inhibitory factor-1, regulate bone homeostasis and serve as treatment targets for bone diseases (65, 67).

MAPK signalling cascades are also activated under mechanical forces and mainly function in bone formation via the up-regulation of many osteogenic genes. Under mechanical stimulus, three major downstream cascades of the MAPK signalling pathway, including extracellular signal-regulated kinase 1/2 (ERK1/2), p38 kinase, and c-Jun N-terminal kinase (JNK), are activated, while osteoblast markers, such as BMP-2, ALP, RUNX2, osteopontin, and osterix, demonstrate enhanced expression (68).

Hippo-YAP is a newly discovered signalling pathway in which YAP/TAZ senses cellular microenvironment changes, such as structural and mechanical alterations. External changes, such as cell–cell contact, cell stretching, and cell shape, lead to actin cytoskeleton remodelling, which controls YAP/TAZ activity. WNT5A and FZD4 may be positively regulated by YAP, and the YAP/WNT5A/FZD4 axis contributes to the osteogenesis of PDLCs under stretch forces (69). Moreover, the Hippo-YAP signalling pathway is responsible for the progression of periodontitis under pathological forces such as traumatic occlusion. Crosstalk between Hippo-Yap and JNK pathways during traumatic occlusion and periodontitis leads to the up-regulation of JNK downstream effector activator protein AP-1 and inflammatory cytokines (IL-6, IL-8, TNF-α, etc.) that stimulate RANKL expression, leading to bone resorption. Therefore, inhibition of YAP can be considered a new target for the treatment of periodontitis with occlusal trauma (70, 71).

Prostaglandin (PG) E2/cyclooxygenase (COX)-2 is another force-sensitive signalling pathway that may have a bidirectional regulatory role in bone metabolism. PGE2 is an important downstream target of the mechanosensitive ion channel Piezo1 and some mechanosensitive pathways, such as MAPK and Wnt pathways (72, 73). Many researchers suggest that a low PGE2 concentration promotes fibronectin synthesis by osteoblasts, while a high PGE2 concentration causes bone resorption by stimulating osteoclast differentiation (74).

Apart from the typical signalling pathways described above, other pathways, such as the IKK-NF-κB, Notch, JAK2/STAT3, and PIEZO1/Ca^2+^/HIF-1α/SLIT3 signalling pathways, are reported to contribute to bone homeostasis under mechanical forces (75–77).

Gasotransmitters, such as H_2_S and NO have also been reported to regulate bone homeostasis. Endogenous H_2_S can be induced by mechanical forces and secreted by PDLCs to regulate bone metabolism. H_2_S tends to promote osteoclastogenesis through its chemoattractant effect on macrophages and regulates osteoblast activity during orthodontic treatment (78). NO, another gas mediator, can regulate multiple cell behaviours in response to mechanical forces. A previous review concluded that NO not only can function alone to regulate osteoclast and osteoblast activity but can also interact with other signalling pathways, such as Wnt/β-catenin, ERK1/2, and PI3K/AKT, to enhance other cell activities (74).

### 2.5 Characteristic PDLSCs function under mechanical forces

PDLSCs exhibit an essential role in PDL tissue maintenance and regeneration. When they are subjected to mechanical forces, the functions of mechanosensing and mechanotransduction enable them to transfer extracellular forces into biological signals that induce cell proliferation, self-renewal, and differentiation (79). Many studies have reported the potential of PDLSCs to promote soft and hard tissue formation. Regulation of PDLSC subpopulations under mechanical forces with regard to periodontal homeostasis has raised much attention in recent years. With the application of the cell lineage tracing technique, subpopulations of PDLSCs characterised with markers, including leptin receptor (Lepr) and Gli, have been identified as contributors to periodontal homeostasis under mechanical forces. Lepr+ cells may be activated by injury and force stimulation via Piezo 1, while Lepr+ stem cells may induce periodontal regeneration following periodontal damage due to pathological forces (8). Further, mechanical forces are essential for the activation of Gli+ multipotential stem cells to periodontal tissues and promote bone remodelling and injury repair (9, 18). In addition, Axin2+ PDL progenitor cells are highly sensitive to tension forces and play an important role in tension force-induced PDL expansion and alveolar bone formation (80). The discovery of PDLSCs with additional characteristics to promote tissue regeneration under specific conditions remains a promising area of research.

### 2.6 Non-coding RNAs

ncRNAs, which account for more than 90% of cellular RNAs, do not code for proteins but instead play regulatory roles in many biological processes involved in cell differentiation, metabolism, and function. ncRNAs are usually divided into two subtypes: short ncRNAs (<200 nucleotides) and long ncRNAs (lncRNAs; >200 nucleotides); microRNAs (miRNAs) are short ncRNAs, whereas lncRNAs exhibit a similar biological origin as mRNA. lncRNA functions in transcriptional, post-transcriptional, and epigenetic regulation of gene expression (81). miRNA, circular RNA (circRNA), and some specific lncRNAs have been reported to be mechanosensitive. miRNA regulates bone metabolism and periodontal homeostasis in response to mechanical forces, and its differential expression depends on the intensity and duration of applied forces. circRNA is a structurally stable lncRNA with a single-stranded covalent closure. A large number (2678) of differentially expressed circRNAs are induced in force-stimulated PDLSCs, while specific circRNAs may promote PDLSC osteogenic differentiation (82, 83). Furthermore, some lncRNAs, such as DANCR, p21, and SNHG8, also function by manipulating signalling pathways and cytokines (84).

At the mechanotransduction stage, ncRNA may increase the mechanical sensitivity of mechanoreceptors and ion channels; ncRNA also interacts with downstream signalling pathways to control force-biology signal conversion. During anabolic and catabolic phases, ncRNA may interact with osteogenesis- and osteoclastogenesis-related transcription factors such as RUNX2 or indirectly change the expression of osteogenic- and osteoclastogenic-associated molecules, such as RANKL-RANK-OPG, Wnt/β-catenin, or TGF-β/BMP to regulate bone metabolism. In addition, ncRNA regulates osteoclast and osteoblast differentiation, maturation, and function through signalling cascades. Apart from bone metabolism, ncRNA is also involved in autophagy, which serves as a kind of adaption in the periodontium under mechanical forces characterised by ECM degradation and reuse. Some therapeutic forces may induce aseptic inflammatory responses in PDLCs; ncRNAs may regulate the inflammatory and immune response by targeting inflammatory cytokines and signal pathways (84).

## 3 Discussion

The concept of periodontal homeostasis, proposed in recent years, provides a new perspective of periodontal health. Periodontal homeostasis refers to the dynamic equilibrium of periodontal tissue metabolism including gingiva metabolism, PDL metabolism and bone metabolism. Gingiva metabolism contributes to the immune barrier of the periodontium. The microbiota-epithelium barrier is quite essential for the periodontal tissues to defend against external stimuli that are important for maintaining tissue immune homeostasis. Besides, mechanical forces could induce immune cells to differentiate into several types, while the type of immune cell induced by pathological forces is often averse to eliminating pathogens and tissue repair. PDL, acted as a “cushion”, could transmit force exerted on teeth to the alveolar bone. Under forces, ECM metabolism involves in the synthesis of PDL fibres as well as angiogenesis that helps maintain PDL integrity. Some certain proteins such as collagen, fibronectin, periostin, fibrillin, and integrin help maintain PDL integrity when pathological or improper therapeutic forces are applied; certain proteins may also participate in repairing damage. Characteristic PDLSCs such as Lepr+, Gli+, and Axin+ cells respond to forces and promote tissue regeneration. Bone metabolism is the ultimate change under applied forces, which manifests as bone formation and resorption. Many mechanosensitive signalling pathways have been found to regulate bone and ECM metabolism while the ncRNAs also function in the soft and hard tissue homeostasis.

Teeth are subjected to physiological or non-physiological mechanical forces. Physiological forces, such as mastication and occlusion, play a protective role in periodontal tissues while the specific mechanisms are concluded above. Mastication not only promotes bone and matrix metabolism but also helps clean teeth. A previous study has found that loss of occlusion forces on teeth resulted in bone resorption and periodontium atrophy (19). Therefore, appropriate forces exerted on teeth are essential for the periodontal tissues. However, there is a particular issue, although teeth are under normal physiological forces, loose teeth caused by periodontitis often experience malocclusion during the occlusal process. Under this circumstance, physiological forces will aggravate periodontitis. Therefore, treating periodontitis in time is essential, fix or extract loose teeth, and adjust occlusion (85). Non-physiological forces include pathological and therapeutic forces. Pathological forces, such as traumatic occlusion and bruxism cause damage to periodontal tissues. When the destructive forces exceed the ability of the periodontal tissue to repair itself, forces would cause cell death through mechanisms, such as apoptosis, pyroptosis, or necrosis, which is characterised by cell membrane rupture and cell content outflow that induces a strong inflammatory response. This implies a breakdown of periodontal homeostasis. However, whether pathological forces alone would cause periodontitis remains to be studied. Normally, proper therapeutic forces such as orthodontic forces promote tooth movement through bone metabolism and PDL metabolism according to the direction of applied force, which influences periodontal homeostasis. The impact of improper therapeutic forces is the same as pathological forces.

In conclusion, we summarise the mechanisms of mechanical forces including physiological forces, pathological forces and therapeutic forces in periodontal homeostasis to help recognise the essential role of force on the periodontal health. We hope that the potential target could be found by clinicians to provide treatment of some periodontal diseases.

## References

[1] Wang S Qin L. Homeostatic medicine: a strategy for exploring health and disease. Curr Med (Cham). (2022) 1:16. doi: 10.1007/s44194-022-00016-9 36189427 PMC9510546

[2] Qin LZ Zhou J Hu L Wang SL. [Homeostatic medicine: new strategy and concept of health maintenance as well as diagnosis and treatment of diseases]. Zhonghua Kou Qiang Yi Xue Za Zhi. (2023) 58:109–17. doi: 10.3760/cma.j.cn112144-20221206-00607 36746443

[3] Loos BG Van Dyke TE. The role of inflammation and genetics in periodontal disease. Periodontol 2000. (2020) 83:26–39. doi: 10.1111/prd.12297 32385877 PMC7319430

[4] Williams DW Greenwell-Wild T Brenchley L Dutzan N Overmiller A Sawaya AP. Human oral mucosa cell atlas reveals a stromal-neutrophil axis regulating tissue immunity. Cell. (2021) 184:4090–104.e15. doi: 10.1016/j.cell.2021.05.013 34129837 PMC8359928

[5] Sedghi L DiMassa V Harrington A Lynch SV Kapila YL. The oral microbiome: Role of key organisms and complex networks in oral health and disease. Periodontol 2000. (2021) 87:107–31. doi: 10.1111/prd.12393 PMC8457218 34463991

[6] Behm C Nemec M Weissinger F Rausch MA Andrukhov O Jonke E. MMPs and TIMPs expression levels in the periodontal ligament during orthodontic tooth movement: A systematic review of *in vitro* and *in vivo* studies. Int J Mol Sci. (2021) 22:6967. doi: 10.3390/ijms22136967 34203475 PMC8268288

[7] Case LB Waterman CM. Integration of actin dynamics and cell adhesion by a three-dimensional, mechanosensitive molecular clutch. Nat Cell Biol. (2015) 17:955–63. doi: 10.1038/ncb3191 PMC6300998 26121555

[8] Zhang D Lin W Jiang S Deng P Liu L Wang Q. Lepr-expressing PDLSCs contribute to periodontal homeostasis and respond to mechanical force by piezo1. Adv Sci (Weinh). (2023) 10:e2303291. doi: 10.1002/advs.202303291 37553778 PMC10582421

[9] Liu AQ Zhang LS Chen J Sui BD Liu J Zhai QM. Mechanosensing by Gli1(+) cells contributes to the orthodontic force-induced bone remodelling. Cell Prolif. (2020) 53:e12810. doi: 10.1111/cpr.12810 32472648 PMC7260067

[10] Qin L Liu W Cao H Xiao G. Molecular mechanosensors in osteocytes. Bone Res. (2020) 8:23. doi: 10.1038/s41413-020-0099-y 32550039 PMC7280204

[11] Diekwisch TGH. Periodontal homeostasis: from vienna to texas-A century of periodontal research in the spirit of bernhard gottlieb. Stem Cells Dev. (2019) 28:961–2. doi: 10.1089/scd.2019.0126 PMC6661909 31218925

[12] Luan X Zhou X Trombetta-eSilva J Francis M Gaharwar AK Atsawasuwan P. MicroRNAs and periodontal homeostasis. J Dent Res. (2017) 96:491–500. doi: 10.1177/0022034516685711 28068481 PMC5453493

[13] Tantilertanant Y Niyompanich J Everts V Supaphol P Pavasant P Sanchavanakit N. Cyclic tensile force-upregulated IL6 increases MMP3 expression by human periodontal ligament cells. Arch Oral Biol. (2019) 107:104495. doi: 10.1016/j.archoralbio.2019.104495 31377584

[14] Li M Zhang C Yang Y. Effects of mechanical forces on osteogenesis and osteoclastogenesis in human periodontal ligament fibroblasts: A systematic review of in *vitro* studies. Bone Joint Res. (2019) 8:19–31. doi: 10.1302/2046-3758.81.BJR-2018-0060.R1 30800296 PMC6359886

[15] Ma Q Ma Z Liang M Luo F Xu J Dou C. The role of physical forces in osteoclastogenesis. J Cell Physiol. (2019) 234:12498–507. doi: 10.1002/jcp.28108 PMC13484134 30623443

[16] Koivumaa KK Mäkilä E Honka O. Histological changes in human periodontium of teeth in masticatory hyper- and hypofunction. Suom Hammaslaak Toim. (1971) 67:122–36. 5284338

[17] Jang AT Chen L Shimotake AR Landis W Altoe V Aloni S. A force on the crown and tug of war in the periodontal complex. J Dent Res. (2018) 97:241–50. doi: 10.1177/0022034517744556 PMC5833186 29364757

[18] Men Y Wang Y Yi Y Jing D Luo W Shen B. Gli1+ Periodontium stem cells are regulated by osteocytes and occlusal force. Dev Cell. (2020) 54:639–54.e6. doi: 10.1016/j.devcel.2020.06.006 32652075

[19] Zhu R Zhang Z Lu B Zhang P Liu W Liang X. Unloading of occlusal force aggravates alveolar bone loss in periodontitis. J Periodontal Res. (2022) 57:1070–82. doi: 10.1111/jre.13047 35973065

[20] Romani P Valcarcel-Jimenez L Frezza C Dupont S. Crosstalk between mechanotransduction and metabolism. Nat Rev Mol Cell Biol. (2020) 22:22–38. doi: 10.1038/s41580-020-00306-w 33188273

[21] Inchingolo AD Di Cosola M Inchingolo AM Greco LucChina A Malcangi G Pettini F. Correlation between occlusal trauma and oral microbiota: a microbiological investigation. J Biol Regul Homeost Agents. (2021) 35:295–302. doi: 10.23812/21-2supp1-29 34281326

[22] Hajishengallis G. New developments in neutrophil biology and periodontitis. Periodontol 2000. (2020) 82:78–92. doi: 10.1111/prd.12313 31850633

[23] Feng Y Fu X Lou X Fu B. Stromal cell-derived factor 1 protects human periodontal ligament stem cells against hydrogen peroxide-induced apoptosis. Mol Med Rep. (2017) 16:5001–6. doi: 10.3892/mmr.2017.7192 28791359

[24] Jiang N He D Ma Y Su J Wu X Cui S. Force-induced autophagy in periodontal ligament stem cells modulates M1 macrophage polarization via AKT signaling. Front Cell Dev Biol. (2021) 9:666631. doi: 10.3389/fcell.2021.666631 34124048 PMC8187804

[25] Lin J Huang J Zhang Z Yu X Cai X Liu C. Periodontal ligament cells under mechanical force regulate local immune homeostasis by modulating Th17/Treg cell differentiation. Clin Oral Investig. (2022) 26:3747–64. doi: 10.1007/s00784-021-04346-0 35029749

[26] Shimohira D Kido MA Danjo A Takao T Wang B Zhang J-Q. TRPV2 expression in rat oral mucosa. Histochem Cell Biol. (2009) 132:423–33. doi: 10.1007/s00418-009-0616-y 19579031

[27] Adelfio M Bonzanni M Callen GE Paster BJ Hasturk H Ghezzi CE. A physiologically relevant culture platform for long-term studies of *in vitro* gingival tissue. Acta Biomaterialia. (2023) 167:321–34. doi: 10.1016/j.actbio.2023.06.008 PMC10528240 37331612

[28] Zhang C Zhang W Zhu D Li Z Wang Z Li J. Nanoparticles functionalized with stem cell secretome and CXCR4-overexpressing endothelial membrane for targeted osteoporosis therapy. J Nanobiotechnol. (2022) 20:35. doi: 10.1186/s12951-021-01231-6 PMC8760699 35033095

[29] Rath-Deschner B Memmert S Damanaki A de Molon RS Nokhbehsaim M Eick S. CXCL5, CXCL8, and CXCL10 regulation by bacteria and mechanical forces in periodontium. Ann Anat. (2021) 234:151648. doi: 10.1016/j.aanat.2020.151648 33221386

[30] Almubarak A Tanagala KKK Papapanou PN Lalla E Momen-Heravi F. Disruption of monocyte and macrophage homeostasis in periodontitis. Front Immunol. (2020) 11:330. doi: 10.3389/fimmu.2020.00330 32210958 PMC7067288

[31] He D Liu F Cui S Jiang N Yu H Zhou Y. Mechanical load-induced H2S production by periodontal ligament stem cells activates M1 macrophages to promote bone remodeling and tooth movement via STAT1. Stem Cell Res Ther. (2020) 11:112. doi: 10.1186/s13287-020-01607-9 32169104 PMC7071778

[32] Balaji S Cholan PK Victor DJ. An emphasis of T-cell subsets as regulators of periodontal health and disease. J Clin Transl Res. (2021) 7:648–56. PMC8580519 34778595

[33] Zhang Y Chen J Fu H Kuang S He F Zhang M. Exosomes derived from 3D-cultured MSCs improve therapeutic effects in periodontitis and experimental colitis and restore the Th17 cell/Treg balance in inflamed periodontium. Int J Oral Sci. (2021) 13:43. doi: 10.1038/s41368-021-00150-4 34907166 PMC8671433

[34] D’Arcy MS. Cell death: a review of the major forms of apoptosis, necrosis and autophagy. Cell Biol Int. (2019) 43:582–92. doi: 10.1002/cbin.11137 30958602

[35] Gudipaty SA Lindblom J Loftus PD Redd MJ Edes K Davey CF. Mechanical stretch triggers rapid epithelial cell division through Piezo1. Nature. (2017) 543:118–21. doi: 10.1038/nature21407 PMC5334365 28199303

[36] Jiang M Shang Z Zhang T Yin X Liang X Sun H. Study on the role of pyroptosis in bone resorption induced by occlusal trauma with or without periodontitis. J Periodontal Res. (2022) 57:448–60. doi: 10.1111/jre.12974 35141913

[37] Pinto VI Senini VW Wang Y Kazembe MP McCulloch CA. Filamin A protects cells against force-induced apoptosis by stabilizing talin- and vinculin-containing cell adhesions. FASEB J. (2014) 28:453–63. doi: 10.1096/fj.13-233759 24097310

[38] Monier B Suzanne M. Orchestration of force generation and nuclear collapse in apoptotic cells. Int J Mol Sci. (2021) 22:10257. doi: 10.3390/ijms221910257 34638598 PMC8508646

[39] Dieterle MP Husari A Steinberg T Wang X Ramminger I Tomakidi P. From the matrix to the nucleus and back: mechanobiology in the light of health, pathologies, and regeneration of oral periodontal tissues. Biomolecules. (2021) 11:824. doi: 10.3390/biom11060824 34073044 PMC8228498

[40] Ma J Zhao D Wu Y Xu C Zhang F. Cyclic stretch induced gene expression of extracellular matrix and adhesion molecules in human periodontal ligament cells. Arch Oral Biol. (2015) 60:447–55. doi: 10.1016/j.archoralbio.2014.11.019 25541636

[41] Chang PE Li S Kim HY Lee DJ Choi YJ Jung HS. BBS7-SHH signaling activity regulates primary cilia for periodontal homeostasis. Front Cell Dev Biol. (2021) 9:796274. doi: 10.3389/fcell.2021.796274 34957122 PMC8703258

[42] Pumklin J Manokawinchoke J Bhalang K Pavasant P. Intermittent compressive stress enhanced insulin-like growth factor-1 expression in human periodontal ligament cells. Int J Cell Biol. (2015) 2015:369874. doi: 10.1155/2015/369874 26106417 PMC4464684

[43] Denes BJ Ait-Lounis A Wehrle-Haller B Kiliaridis S. Core matrisome protein signature during periodontal ligament maturation from pre-occlusal eruption to occlusal function. Front Physiol. (2020) 11. doi: 10.3389/fphys.2020.00174 PMC7066325 32194440

[44] Wei L Chen Q Zheng Y Nan L Liao N Mo S. Potential role of integrin α_5_β_1_/focal adhesion kinase (FAK) and actin cytoskeleton in the mechanotransduction and response of human gingival fibroblasts cultured on a 3-dimension lactide-co-glycolide (3D PLGA) scaffold. Med Sci Monit. (2020) 26:e921626. doi: 10.12659/MSM.921626 32034900 PMC7027369

[45] Ye Y Zhang R Feng H. Fibronectin promotes tumor cells growth and drugs resistance through a CDC42-YAP-dependent signaling pathway in colorectal cancer. Cell Biol Int. (2020) 44:1840–9. doi: 10.1002/cbin.11390 32437085

[46] Yuan C Li J. Research progress of periostin and osteoporosis. Front Endocrinol (Lausanne). (2024) 15:1356297. doi: 10.3389/fendo.2024.1356297 38487345 PMC10938139

[47] Rios H Koushik SV Wang H Wang J Zhou HM Lindsley A. periostin null mice exhibit dwarfism, incisor enamel defects, and an early-onset periodontal disease-like phenotype. Mol Cell Biol. (2005) 25:11131–44. doi: 10.1128/MCB.25.24.11131-11144.2005 PMC1316984 16314533

[48] Panchamanon P Pavasant P Leethanakul C. Periostin plays role in force-induced stem cell potential by periodontal ligament stem cells. Cell Biol Int. (2019) 43:506–15. doi: 10.1002/cbin.11116 30761669

[49] Na Nan D Klincumhom N Trachoo V Everts V Ferreira JN Osathanon T. Periostin-integrin interaction regulates force-induced TGF-β1 and α-SMA expression by hPDLSCs. Oral Dis. (2023). 30:2570–9. doi: 10.1111/odi.14691 37466141

[50] Tanabe H Takayama I Nishiyama T Shimazaki M Kii I Li M. Periostin associates with Notch1 precursor to maintain Notch1 expression under a stress condition in mouse cells. PloS One. (2010) 5:e12234. doi: 10.1371/journal.pone.0012234 20805882 PMC2923609

[51] Padial-Molina M Volk SL Taut AD Giannobile WV Rios HF. Periostin is down-regulated during periodontal inflammation. J Dent Res. (2012) 91:1078–84. doi: 10.1177/0022034512459655 PMC3525130 22933606

[52] Shin SJ Yanagisawa H. Recent updates on the molecular network of elastic fiber formation. Essays Biochem. (2019) 63:365–76. doi: 10.1042/EBC20180052 31395654

[53] Yoshikazu M Shiga M Kometani-Gunjigake K Nakao-Kuroishi K Mizuhara M Toyono T. Fibrillin-1 regulates periostin expression during maintenance of periodontal homeostasis. J Dent Sci. (2022) 17:1714–21. doi: 10.1016/j.jds.2022.02.015 PMC9588790 36299324

[54] Kanchanawong P Calderwood DA. Organization, dynamics and mechanoregulation of integrin-mediated cell–ECM adhesions. Nat Rev Mol Cell Biol. (2022) 24:142–61. doi: 10.1038/s41580-022-00531-5 PMC9892292 36168065

[55] Dieterle MP Husari A Steinberg T Wang X Ramminger I Tomakidi P. Role of mechanotransduction in periodontal homeostasis and disease. J Dent Res. (2021) 100:1210–9. doi: 10.1177/00220345211007855 33870741

[56] Kechagia JZ Ivaska J Roca-Cusachs P. Integrins as biomechanical sensors of the microenvironment. Nat Rev Mol Cell Biol. (2019) 20:457–73. doi: 10.1038/s41580-019-0134-2 31182865

[57] Bays JL Campbell HK Heidema C Sebbagh M DeMali KA. Linking E-cadherin mechanotransduction to cell metabolism through force-mediated activation of AMPK. Nat Cell Biol. (2017) 19:724–31. doi: 10.1038/ncb3537 PMC5494977 28553939

[58] Beckmann D Römer-Hillmann A Krause A Hansen U Wehmeyer C Intemann J. Lasp1 regulates adherens junction dynamics and fibroblast transformation in destructive arthritis. Nat Commun. (2021) 12:3624. doi: 10.1038/s41467-021-23706-8 34131132 PMC8206096

[59] Feng L Zhang Y Kou X Yang R Liu D Wang X. Cadherin-11 modulates cell morphology and collagen synthesis in periodontal ligament cells under mechanical stress. Angle Orthod. (2017) 87:193–9. doi: 10.2319/020716-107.1 PMC8384358 27689865

[60] Uhler C Shivashankar GV. Regulation of genome organization and gene expression by nuclear mechanotransduction. Nat Rev Mol Cell Biol. (2017) 18:717–27. doi: 10.1038/nrm.2017.101 29044247

[61] Maurer M Lammerding J. The driving force: nuclear mechanotransduction in cellular function, fate, and disease. Annu Rev BioMed Eng. (2019) 21:443–68. doi: 10.1146/annurev-bioeng-060418-052139 PMC6815102 30916994

[62] Vahabikashi A Adam SA Medalia O Goldman RD. Nuclear lamins: Structure and function in mechanobiology. APL Bioeng. (2022) 6:011503. doi: 10.1063/5.0082656 35146235 PMC8810204

[63] Han Y You X Xing W Zhang Z Zou W. Paracrine and endocrine actions of bone-the functions of secretory proteins from osteoblasts, osteocytes, and osteoclasts. Bone Res. (2018) 6:16. doi: 10.1038/s41413-018-0019-6 29844945 PMC5967329

[64] Geoghegan IP Hoey DA McNamara LM. Integrins in osteocyte biology and mechanotransduction. Curr Osteoporos Rep. (2019) 17:195–206. doi: 10.1007/s11914-019-00520-2 31250372

[65] Baron R Kneissel M. WNT signaling in bone homeostasis and disease: from human mutations to treatments. Nat Med. (2013) 19:179–92. doi: 10.1038/nm.3074 23389618

[66] Xu W Lu Q Qu M Fan R Leng S Wang L. Wnt4 regulates bone metabolism through IKK-NF-κB and ROCK signaling under occlusal traumatic periodontitis. J Periodontal Res. (2022) 57:461–9. doi: 10.1111/jre.12975 35137408

[67] Manokawinchoke J Limjeerajarus N Limjeerajarus C Sastravaha P Everts V Pavasant P. Mechanical force-induced TGFB1 increases expression of SOST/POSTN by hPDL cells. J Dent Res. (2015) 94:983–9. doi: 10.1177/0022034515581372 25870205

[68] Yong J Groeger S Meyle J Ruf S. MAPK and β-Catenin signaling: implication and interplay in orthodontic tooth movement. Front Biosci (Landmark Ed). (2022) 27:54. doi: 10.31083/j.fbl2702054 35226997

[69] Zhang X Chang M Wang B Liu X Zhang Z Han G. YAP/WNT5A/FZD4 axis regulates osteogenic differentiation of human periodontal ligament cells under cyclic stretch. J Periodontal Res. (2023) 58:907–18. doi: 10.1111/jre.13143 37340863

[70] Pan W Yang L Li J Xue L Wei W Ding H. Traumatic occlusion aggravates bone loss during periodontitis and activates Hippo-YAP pathway. J Clin Periodontol. (2019) 46:438–47. doi: 10.1111/jcpe.13065 30629753

[71] Wei W Xue L Tan L Liu J Yang Q Wang J. Inhibition of yes-associated protein dephosphorylation prevents aggravated periodontitis with occlusal trauma. J Periodontol. (2021) 92:1036–48. doi: 10.1002/JPER.19-0338 33094479

[72] Suzuki R Nemoto E Shimauchi H. Cyclic tensile force up-regulates BMP-2 expression through MAP kinase and COX-2/PGE2 signaling pathways in human periodontal ligament cells. Exp Cell Res. (2014) 323:232–41. doi: 10.1016/j.yexcr.2014.02.013 24561081

[73] Robinson JA Chatterjee-Kishore M Yaworsky PJ Cullen DM Zhao W Li C. Wnt/beta-catenin signaling is a normal physiological response to mechanical loading in bone. J Biol Chem. (2006) 281:31720–8. doi: 10.1016/S0021-9258(19)84086-3 16908522

[74] Yan T Xie Y He H Fan W Huang F. Role of nitric oxide in orthodontic tooth movement (Review). Int J Mol Med. (2021) 48:168. doi: 10.3892/ijmm 34278439 PMC8285047

[75] Chen Y Yin Y Luo M Wu J Chen A Deng L. Occlusal force maintains alveolar bone homeostasis via type H angiogenesis. J Dent Res. (2023) 102:1356–65. doi: 10.1177/00220345231191745 37786932

[76] Jin Y Ding L Ding Z Fu Y Song Y Jing Y. Tensile force-induced PDGF-BB/PDGFRβ signals in periodontal ligament fibroblasts activate JAK2/STAT3 for orthodontic tooth movement. Sci Rep. (2020) 10:11269. doi: 10.1038/s41598-020-68068-1 32647179 PMC7347599

[77] Kikuta J Yamaguchi M Shimizu M Yoshino T Kasai K. Notch signaling induces root resorption via RANKL and IL-6 from hPDL cells. J Dent Res. (2015) 94:140–7. doi: 10.1177/0022034514555364 25376720

[78] Liu F Wen F He D Liu D Yang R Wang X. Force-induced H(2)S by PDLSCs modifies osteoclastic activity during tooth movement. J Dent Res. (2017) 96:694–702. doi: 10.1177/0022034517690388 28165889

[79] Vining KH Mooney DJ. Mechanical forces direct stem cell behaviour in development and regeneration. Nat Rev Mol Cell Biol. (2017) 18:728–42. doi: 10.1038/nrm.2017.108 PMC5803560 29115301

[80] Wang K Xu C Xie X Jing Y Chen PJ Yadav S. Axin2+ PDL cells directly contribute to new alveolar bone formation in response to orthodontic tension force. J Dent Res. (2022) 101:695–703. doi: 10.1177/00220345211062585 35001706 PMC9124907

[81] Slack FJ Chinnaiyan AM. The role of non-coding RNAs in oncology. Cell. (2019) 179:1033–55. doi: 10.1016/j.cell.2019.10.017 PMC7347159 31730848

[82] Wang H Feng C Jin Y Tan W Wei F. Identification and characterization of circular RNAs involved in mechanical force-induced periodontal ligament stem cells. J Cell Physiol. (2019) 234:10166–77. doi: 10.1002/jcp.27686 30422310

[83] Wang Y Jia L Zheng Y Li W. Bone remodeling induced by mechanical forces is regulated by miRNAs. Biosci Rep. (2018) 38:BSR20180448. doi: 10.1042/BSR20180448 29844019 PMC6028748

[84] Chen Y Zhang C. Role of noncoding RNAs in orthodontic tooth movement: new insights into periodontium remodeling. J Transl Med. (2023) 21:101. doi: 10.1186/s12967-023-03951-9 36759852 PMC9912641

[85] Passanezi E Sant’Ana ACP. Role of occlusion in periodontal disease. Periodontol 2000. (2019) 79:129–50. doi: 10.1111/prd.12251 30892765

